# Role of substrate temperature on the performance of BaTiO_3_/Si photodetector prepared by pulsed laser deposition

**DOI:** 10.1038/s41598-024-55053-1

**Published:** 2024-02-24

**Authors:** Nadheer Z. Abed, Raid A. Ismail, Suaad S. Shaker

**Affiliations:** Applied Science Department, University of Technology, Baghdad, Iraq

**Keywords:** BaTiO_3_, Pulsed laser deposition, Nanostructure film, Substrate temperature, Photodetector, Materials for devices, Nanoscale materials

## Abstract

In this study, the pulsed laser deposition (PLD) method was employed to fabricate nanostructured BaTiO_3_ films on glass and silicon substrates at varying temperatures. The structural analysis confirmed the formation of crystalline nanostructured BaTiO_3_ with mixed tetragonal and hexagonal phases, and the film deposited at 150 °C has the best crystallinity and largest particle size**.** The optical energy gap of the BaTiO_3_ nanostructure decreases from 3.94 to 3.84 eV, with increasing substrate temperature from 60 to 150 °C. Photoluminescence spectra of BaTiO_3_ films deposited at 25, 60, 100, and 150 °C exhibit emission peaks centered at 450, 512, 474, and 531 nm, respectively. Raman spectra of BaTiO_3_ films show E (LO), A (TO), E (LO) + TO, and B1 vibration modes. Hall measurements reveal that the mobility of the BaTiO_3_ film increases with temperature up to 100 °C and then decreases at 150 °C. The current–voltage characteristics of the BaTiO_3_/p-Si heterojunction, deposited over a temperature range of 25 to 150 °C, were investigated in the dark and under illumination. The heterojunctions exhibit rectifying properties, with the best rectification factor observed for the heterojunction prepared at 100 °C**.** The values of the ideality factor for the heterojunctions fabricated at 25, 60, 100, and 150 °C were 4.3, 3.8, 2.8, and 5, respectively. The study reveals an improvement in both the figures of merit and the photodetector performance with increased substrate temperature. The responsivity increases from 2.2 to 9.25 A/W as the deposition temperature rises from 25 to 100 °C. The detectivity (D*) and external quantum efficiency (EQE) of the photodetector prepared at the optimum substrate temperature of 100 °C, were found to be 4.62 × 10^12^ Jones and 114%, respectively, at 500 nm.

## Introduction

Barium titanate (BaTiO_3_) is a significant functional semiconductor within the perovskite class, renowned for its outstanding pyroelectric, ferromagnetic, high mechanical and chemical stability, as well as electro-optic properties. These attributes render it well-suited for diverse industrial and technological uses^1^. BaTiO_3_ displays tetragonal symmetry, marked by the displacement of titanium and oxygen in opposite directions along the elongated axis (c-axis)^2^. Its applications span a broad spectrum, including but not limited to piezoelectric infrared sensors, capacitors, ultrasonic transducers, dye-sensitized solar cells, ferroelectric random-access memories (FRAMs), electro-optic switches, waveguides, thermistors, and actuators^3–5^.

The choice of synthesis technique depends on the desired properties for the final application, and the selected preparation route has a significant impact on the structure and properties of barium titanate materials^6,7^. Various methods are employed for the synthesis of the BT system, including sol–gel^8,9^, solid-state^10^, coprecipitation^11^, chemical vapor deposition (CVD)^12^, hydrothermal^13,14^, electrophoretic deposition^15^, physical radio frequency sputtering^16^, laser molecular beam epitaxy (MBE), flash evaporation, and pulsed laser deposition (PLD)^17–19^.

Pulsed-laser deposition (PLD) is a unique method that distinguishes itself and appears as one of the most encouraging approaches for producing thin films using multicomponent materials. Its merits encompass simplicity, cost-effectiveness, the capability for large-area film deposition, the maintenance of precise film stoichiometry, the attainment of excellent crystallinity in deposited films, and rapid processing. Noteworthy among the extensively investigated materials are high-temperature superconductors, compound semiconductors, dielectrics, ferroelectrics, electro-optic and extremely large magneto-resistance oxides, polymers, and diverse heterostructures. PLD has notably been applied in the deposition of thin films, such as BaTiO_3_. Recently, Serralta-Macías et al.^20^ reported the preparation of lead-free antiferroelectric perovskite ultrathin films (_0.92_(Bi_0.54_Na_0.46_)TiO_3_-0.08BaTiO_3_) on p-type silicon using the pulsed laser deposition technique. Silicon-based heterojunction photodetectors exhibit very attractive optoelectronic and photovoltaic properties compared to p-n silicon photodiodes. They have high responsivity, fast response, low noise, long-wavelength detection, a simple route, cost-effectiveness, and no high-temperature processing needed^21^. Several high-performance silicon-based heterojunction photodetectors have been produced using pulsed-laser deposition (PLD) methods. To the best of our knowledge, there is no reported data on the fabrication of a BaTiO_3_/Si heterojunction photodetector. In this context, we fabricated a BaTiO_3_/Si heterojunction photodetector using the pulsed laser deposition method, omitting the use of a buffer layer. In order to enhance the photodetector's performance, we conducted an investigation into the impact of substrate temperature on the structural, optical, and electrical properties of the BaTiO_3_ film. Additionally, we evaluated the figures of merit associated with the BaTiO3/Si photodetector.

## Experimental work

**Figure 1:**
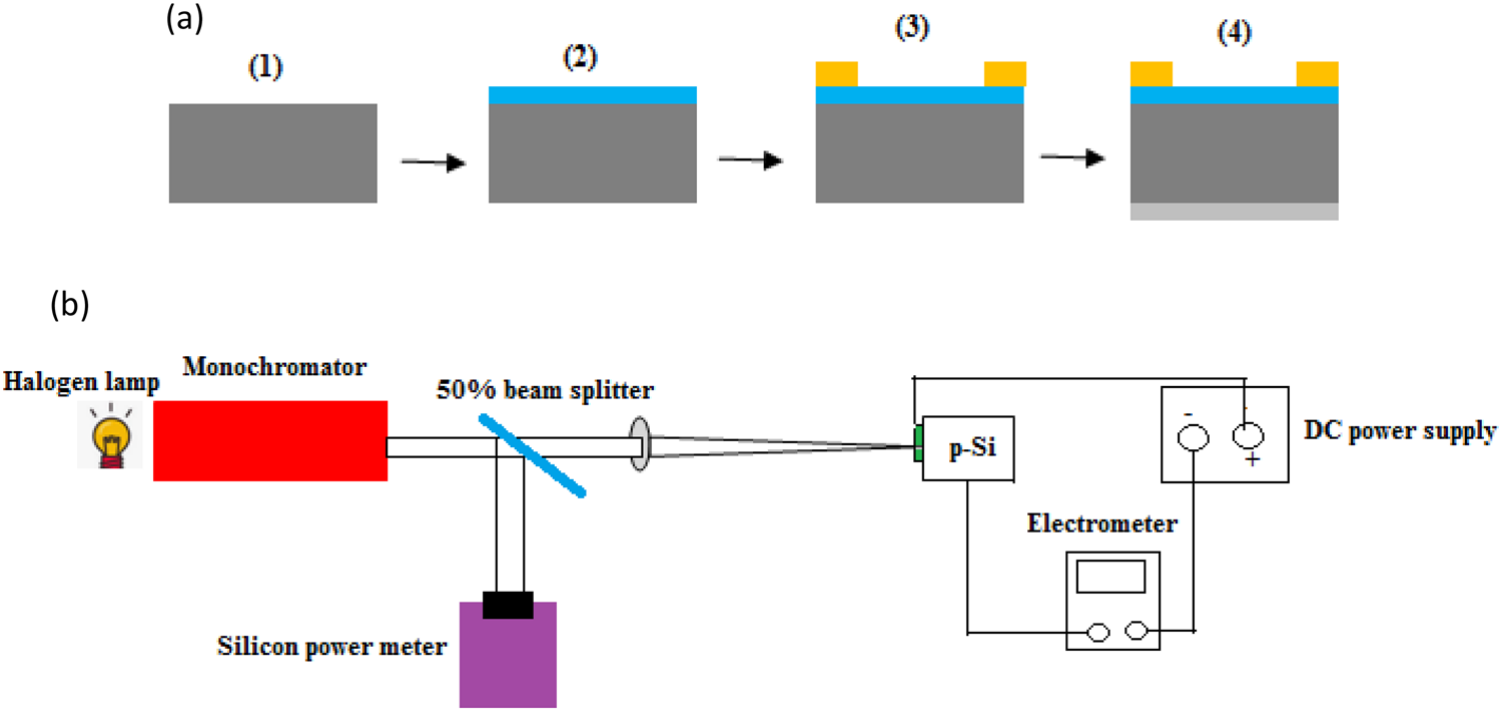
(**a**) Fabrication steps of BaTiO_3_/Si photodetector (1) Cleaned silicon substrate, (2) deposition of BaTiO_3_ film, (3) evaporation of In- electrode on BaTiO_3_ film, and (4) Evaporation of Al-electrode on backside of Si and (**b**) Schematic diagram of dark, illuminated I–V characteristics and responsivity measurements setup.

A homemade pulsed laser deposition (PLD) system was utilized, comprising a Q-switched Nd:YAG laser operating at a wavelength of 532 nm (second harmonic) with a pulse duration of 7 ns, along with a glass bell jar. This system was employed to deposit BaTiO3 thin films onto cleaned glass and silicon substrates. The BaTiO_3_ pellet, measuring 1 cm^2^ in diameter and 2 mm in depth, was created by compressing high-purity (99.99%) BaTiO_3_ powder obtained from Sigma-Aldrich using a hydraulic compressor at 5 tons. The distance between the target and substrate was consistently set at 3 cm for all experiments. The BaTiO_3_ films were deposited by irradiating the BaTiO3 pellet with an energy density of 8.9 J/cm^2^ and 200 pulses under a vacuum pressure of 10^–4^ mbar. The films were deposited on substrates heated to various temperatures: 25, 60, 100, and 150 °C. The glass substrate was initially cleaned with distilled water and alcohol. A polished single crystal 1 cm^2^ p-type silicon substrate with an orientation of (111) and electrical resistivity of 3–5 Ω cm was used after being cleaning with distilled water and treatment with diluted HF. The film structure was analyzed using a Shimadzu XRD-6000 X-ray diffractometer. Atomic force microscopy (AFM), specifically the Digital Instruments Nanoscope II Scanning Probe Microscope (AFM/SPM DualScopeTM DS/Germany), was employed to investigate the surface morphology and particle size distribution of the deposited films. A field emission scanning electron microscope (FESEM) equipped with energy-dispersive X-ray (EDX) from Imaging-EDS-Mapping/Germany was used to examine the chemical composition of the BaTiO_3_ thin film. For optical properties measurements, a double-beam UV–Vis spectrophotometer (UV-DRS Shimadzu UV-2550) was used. Raman shift analysis was conducted with a Raman Spectroscopy system (Raman Malvern HORIBA XploRA PLUS/UK). Photoluminescence measurements were carried out at room temperature using an argon ion laser with a wavelength of 320 nm (PL Varian CARY ECLIPSE) as the excitation source. Hall effect measurements were performed to determine the electrical conductivity and mobility of the deposited films. Ohmic contacts were established by depositing In film on the BaTiO_3_ film and Al electrode on the backside of the silicon substrate through a square thin metal mask using a thermal evaporation system. Figure 1a illustrates the schematic diagram of the BaTiO_3_/Si heterojunction photodetector. The film thickness deposited on silicon substrates ranged from 600 to 1400 nm, depending on the substrate temperature. The current–voltage characteristics of the BaTiO_3_/Si heterojunction were measured under dark and white illumination conditions. The responsivity of the BaTiO_3_/Si photodetector was measured in the spectral range of 400–900 nm using a Jobin Yvon monochromator, beam splitter, and halogen lamp under reverse bias voltage. The power of the light at a certain wavelength was calibrated with a silicon power meter. Figure 1b shows the schematic diagram of the setup used to measure the dark, illuminated I-V characteristics, and spectral responsivity.

## Results and discussion

**Figure 2:**
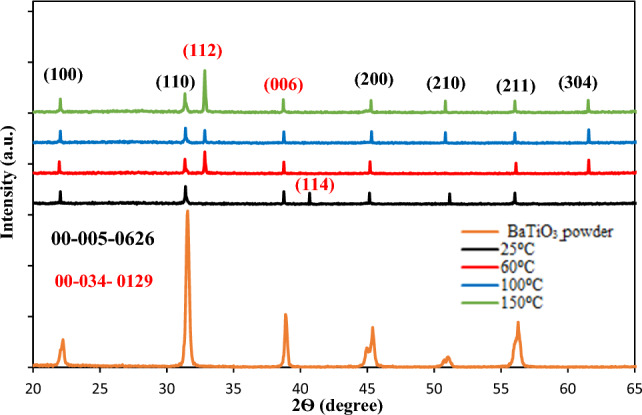
X-ray diffraction patterns of BaTiO_3_ powder and BaTiO_3_ nanostructure deposited at various substrate temperatures.

The XRD patterns of the BaTiO_3_ pellet and films deposited at 25, 60, 100, and 150 °C are illustrated in Fig. 2. The film deposited at 25 °C exhibits seven peaks that confirm the formation of a hexagonal-tetragonal co-existing BaTiO_3_ film. These peaks are observed at 2θ = 22°, 31.4°, 38.9°, 40.52°, 45.1°, 51.1°, and 56°, corresponding to the (100), (110), (006), (114), (200), (210), and (211) reflection planes, respectively. According to JCPDs # 00-005-0626^22^ and 00-034-0129^23^, the BaTiO_3_ film deposited at room temperature (RT) was crystalline and exhibited seven diffraction peaks corresponding to the (100), (110), (006), (114), (200), (210), and (211) reflection planes, respectively. These peaks along (100), (110), (200), (210), and (211) belong to crystalline BaTiO_3_ with a tetragonal phase, while the peaks along (006) and (114) planes indeed belong to hexagonal BaTiO_3_. A small shift in diffraction angles of the XRD peaks of the nanostructured films compared to the bulk is due to the presence of stacking fault^24,25^.

The lattice constant of tetragonal BaTiO_3_ was calculated and found to be a = b = 0.4032 nm, and the c/a ratio was 0.997 nm, which is close to the lattice constant for bulk tetragonal BaTiO3 (a = b = 0.3994 nm) with a c/a ratio of 1.011 nm, according to JCPDs # 00-005-0626. The lattice constants of hexagonal BaTiO_3_ were a = b = 0.57116 nm, and c = 1.3931 nm. The ratio of c/a is 2.4391. These lattice parameters and the c/a ratio are in good agreement with those for bulk BaTiO_3_: a = b = 0.57248 nm, c = 1.39673 nm, and c/a ratio of 2.4397, according to JCPDs # 00-034-0129. Increasing the deposition temperature results in the presence of a new peak found at 32.8° corresponding to the (112) plane, as well as a slight increase in the intensity of the other XRD peaks. This indicates that the crystallinity of the nanostructured film improved after increasing the substrate temperature. This improvement could be attributed to the fact that the kinetic energy of BaTiO_3_ nanoparticles increases as the substrate temperature rises, giving them the ability to move very quickly across the film surface and create low surface energy structures^26^. No XRD peaks related to other elements or contaminants have been found in the XRD patterns, indicating the purity of the deposited nanostructured films. The average crystallite size of the BaTiO_3_ film was estimated using the Debye–Scherer formula:

1 $$D=\frac{0.9\lambda }{\beta cos\theta }$$

where λ is the Cu Kα X-ray wavelength (λ = 0.154 nm), θ is the diffraction angle, and β is the is the full width at half maximum FWHM of the XRD peak. Table 1 lists the values of the average crystallite size of the films deposited at various substrate temperatures.

**Table 1:** Effect of substrate temperature on crystallite size, strain, and dislocation of the film.

Substrate temperature (°C)	Lattice constants for tetragonal structure (nm)			Lattice constants for hexagonal structure (nm)			Crystallite size (D) (nm)	Strain (ɛ)	Dislocation density (nm^-2^)
	a = b	c	c/a	a = b	c	c/a			
25	0.403	0.402	0.997	0.571	1.3931	2.409	28.6	3.05 × 10^–3^	1.22 × 10^–3^
60	0.404	0.401	0.992	0.578	1.3934	2.406	42.8	2.44 × 10^–3^	5.46 × 10^–4^
100	0.403	0.401	0.992	0.578	1.3937	2.407	45.0 5	3.05 × 10^–3^	4.83 × 10^–4^
150	0.403	0.403	0.999	0.578	1.395	2.4107	55.6	3.67 × 10^–3^	3.23 × 10^–4^

The crystallite size increased when the deposition temperature increased as a result of the enhancement of film crystallinity. The strain ε and dislocation density δ formed in the film were calculated using the following equations:

2 $$ \varepsilon = \frac{\beta \cos \theta }{4} $$

3 $${\varvec{\updelta}}=\frac{1}{{D}^{2}}$$

**Figure 3:**
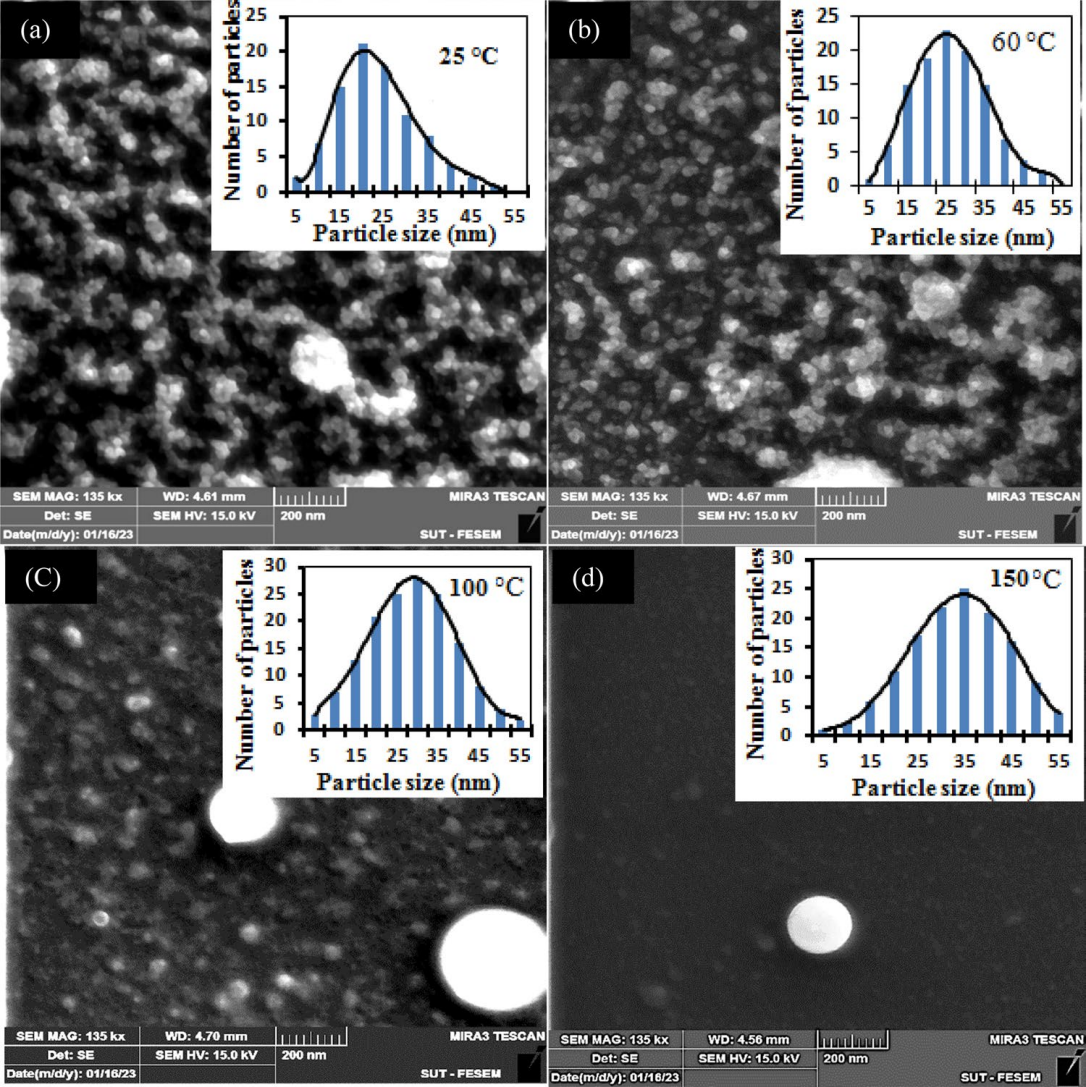
FESEM images of BaTiO_3_ nanoparticles deposited at substrate temperature of (**a**) 25, (**b**) 60, (**c**) 100, and (**d**) 150 °C. Inset are the particle size distribution of the samples.

**Figure 4:**
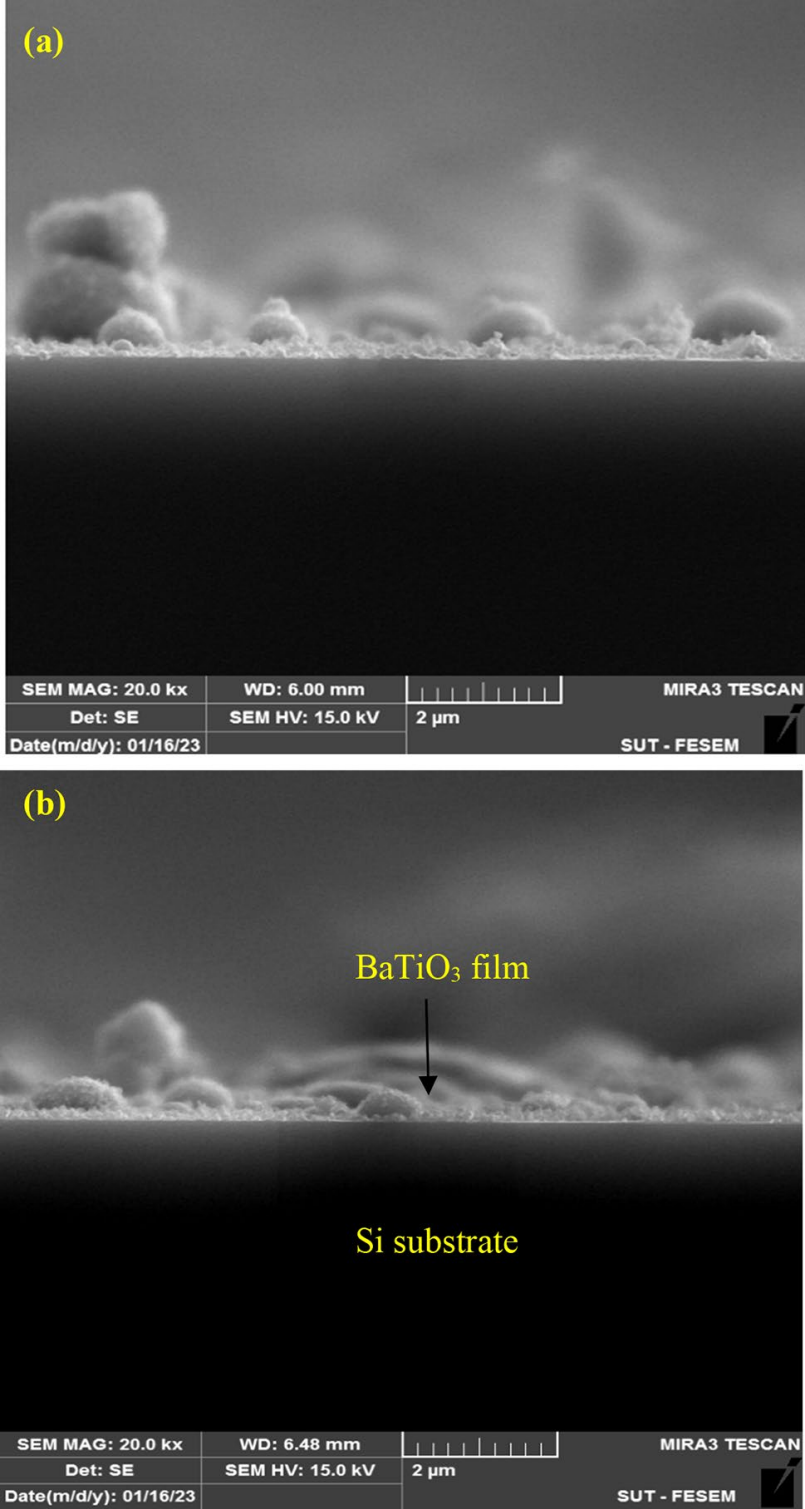
Cross section FESEM images of BaTiO_3_ nanostructures deposited at (**a**) 25 and (**b**) 100 °C.

It can be seen from Table 1 that the strain and dislocation density in the film reduce as the deposition temperature rises due to the decreasing density of the structural defects accompanied by the Fig. 3 shows the FESEM images of the nanostructured BaTiO_3_ films deposited at various substrate temperatures^27^. The film deposited at 25 °C, as shown in Fig. 3a, exhibits the formation of randomly distributed high density of nanoparticles with different sizes, with most grains having a spherical shape. Some larger agglomerated nanoparticles are observed to be formed on the surface, representing the last deposited grains. The average grain size was estimated using Image J software and found to be 16 nm. The film deposited at 60 °C (Fig. 3b) also shows the formation of a high density of nanoparticles, but with a small number of agglomerated particles, and the average particle size was 23 nm. Films deposited at substrate temperatures of 100 °C, as shown in Fig. 3c, exhibit larger grains with an average size of 44 nm, as well as the formation of particulates and some white color droplets due to the laser splashing effect. The film deposited at 150 °C was compact, dense, thicker, and have droplets with an average grain size of 91 nm, as shown in Fig. 3d. Increasing the substrate temperature leads to an increase in the amount of material ablated and deposited on the substrate. Furthermore, the adhesion of the nanostructured film was checked by scratching technique and found to be improved compared to that deposited at room temperature. No cracks or voids have been observed on the film surface. Figure 4 shows the cross section FESEM image of the BaTiO_3_ film deposited on a silicon substrate at substrate temperatures of 25 and 100 °C. The inset of Fig. 4 is the particle size distribution of the samples deposited at various temperatures. The samples have a narrow and nearly Gaussian distribution.

**Figure 5:**
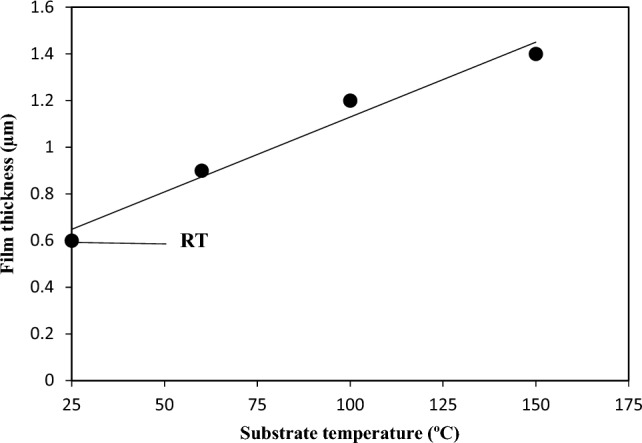
Variation of film thickness with substrate temperature.

The images confirm the formation of spherical nanoparticles and a clear boundary between the film and silicon substrate. The nanostructured film thickness was measured and found to increase with substrate temperature, as shown in Fig. 5. This increase could be due to the increasing ablated and deposited grains with an increase in substrate temperature. As shown in Fig. 5, the thickness of the nanostructured film demonstrated a linear increase with substrate temperature. The calculated increment in film thickness per degree Celsius of substrate temperature was found to be 6.4 nm/°C.

**Figure 6:**
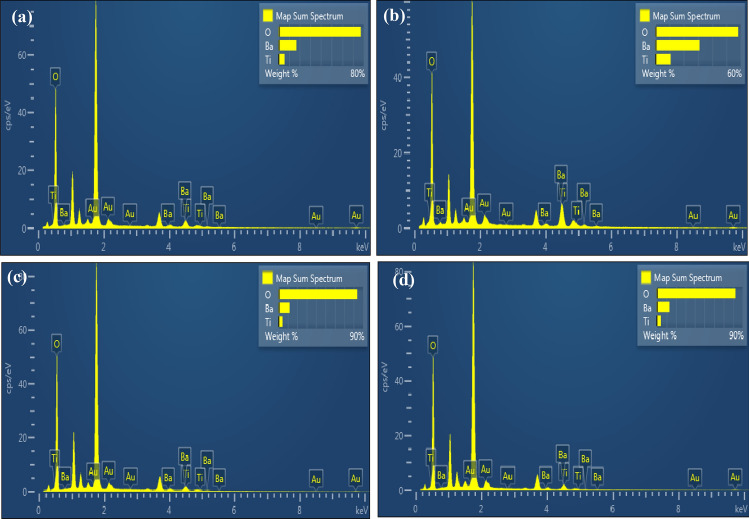
EDX spectra of BaTiO_3_ nanostructure films deposited at (**a**) 25, (**b**) 60, (**c**) 100, and (**d**) 150 °C.

Figure 6 shows the EDX spectra of the BaTiO_3_ film deposited at various substrate temperatures. All spectra confirm the presence of peaks related to the Ba, Ti, and O elements, which are the main elements of the BaTiO_3_ film. The Au peaks come from the deposition of the film on the BaTiO_3_ film for SEM investigation. The weight percentages of the elements are shown in Table 2. The best stoichiometry was found for the film deposited at 60 °C. No other elements were detected in the EDX spectra. Decreasing the amount of barium with the rise in substrate temperature, due to the thermal decomposition of barium as well as the vaporization of the barium component more than the other components, could lead to a barium-deficient film.

**Table 2:** Weight percentage of BaTiO_3_ film as a function of substrate temperature.

Element	Weight%			
	25°C	60°C	100°C	150°C
O_2_	77.2	58.2	83	82.5
Ti	5.8	10.6	4.3	4.5
Ba	16	30.8	11.8	13

**Figure 7:**
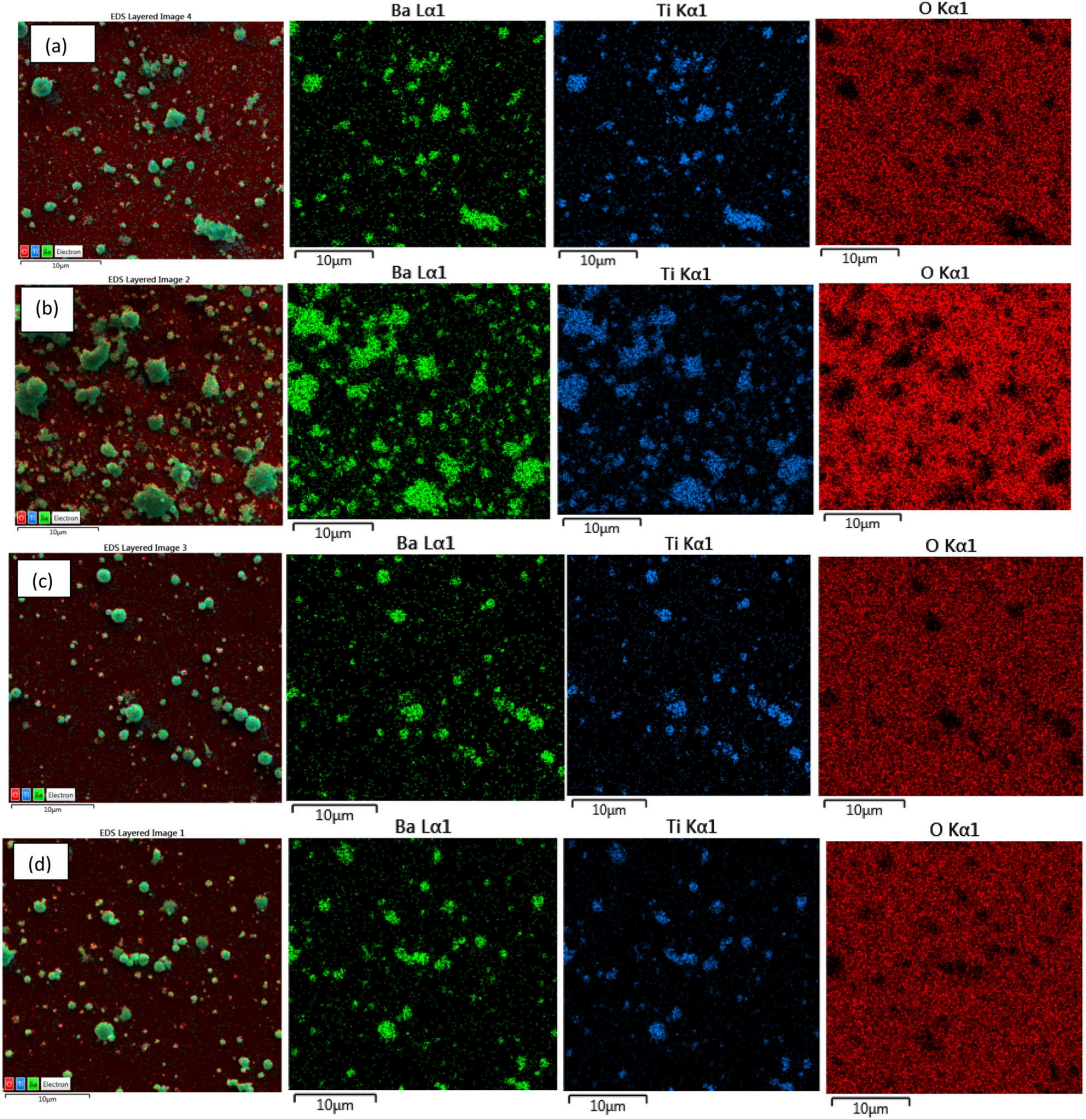
Elemental EDX mapping images of BaTiO_3_ films deposited at substrate temperature of at (**a**) 25 °C (**b**) 60 °C (**c**) 100 °C, and (**d**) 150 °C.

Figure 7 illustrates the EDX mapping images of the films deposited at various substrate temperatures, revealing that the Ba, Ti, and O elements are distributed across the film surface in all samples. The film deposited at 60 °C shows more grain agglomeration compared to the other samples***.***

**Figure 8:**
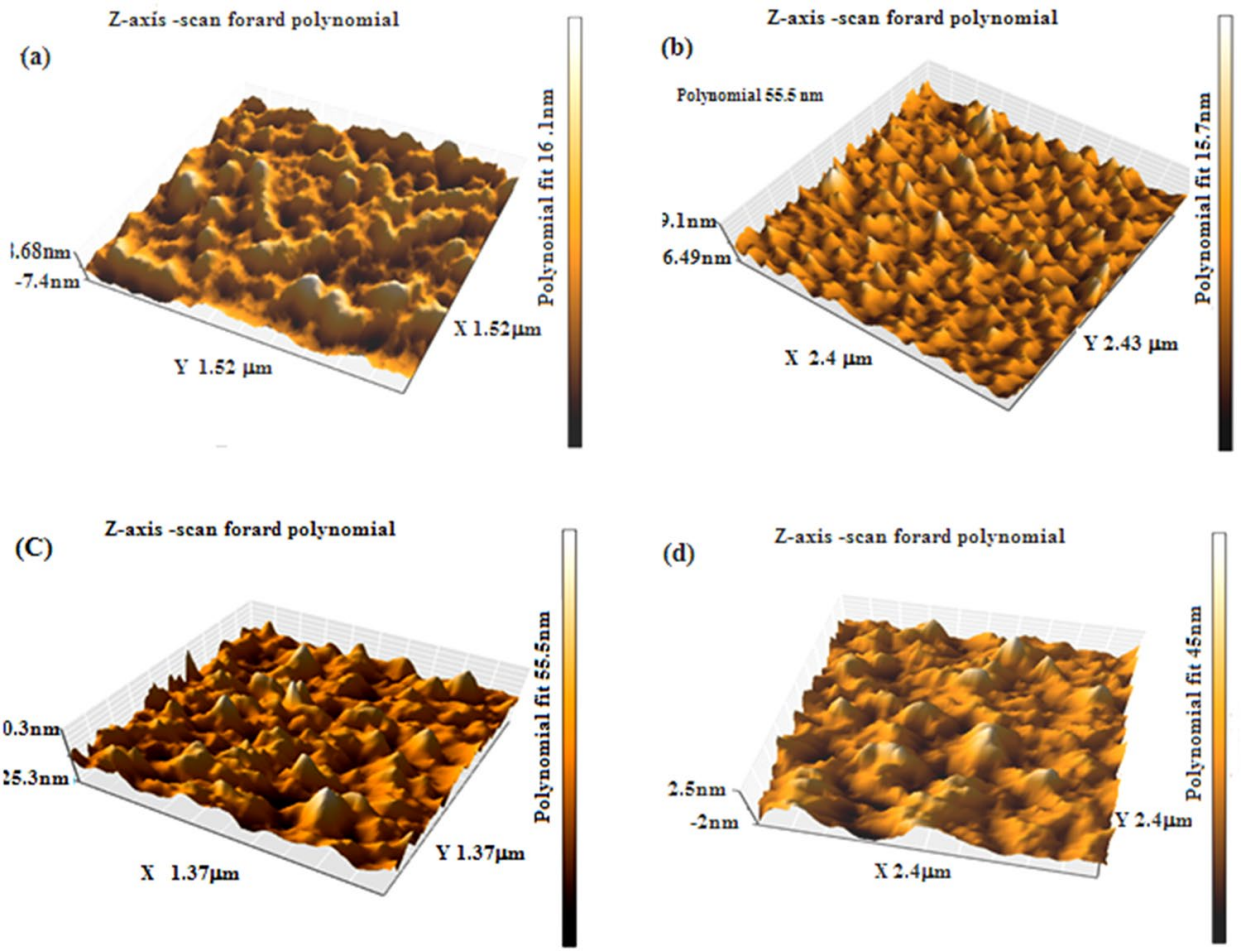
3D AFM images of BaTiO_3_ films deposited at (**a**) 25, (**b**) 60, (**c**) 100 °C, and (**d**) 150 °C.

Figure 8 shows 3D AFM images of the BaTiO_3_ films. The film topography depended strongly on the substrate temperature. The film deposited at room temperature (Fig. 8a) shows the presence of rounded grains, as depicted in Fig. 8b, distributed over the entire surface.

The AFM image of the film deposited at Ts = 60 °C reveals the formation of triangular, uniformly distributed grains with different heights, and the root mean square (RMS) of surface roughness is higher than that of the film deposited at room temperature, as shown in Table 3. Increasing the substrate temperature to 100 60 °C leads to the formation of a small amount of large triangular grains and irregular grains on the film surface. Finally, the AFM image of the film deposited at Ts = 150 60 °C shows large irregular and rounded grains with some triangular grains. The RMS of surface roughness increases with substrate temperature, as shown in Table 3, due to the high kinetic energy and mobility of the deposited adsorbed grains on the substrate^22,28^. The grain size of the film increases with substrate temperature, which is in good agreement with XRD data.

**Table 3:** RMS of surface roughness of the film and grain size obtained from AFM analysis.

Ts (°C)	RMS of roughness (nm)	Grain size (nm)
25	11.17	36
60	19.91	50
100	22.37	68
150	30.50	74

**Figure 9:**
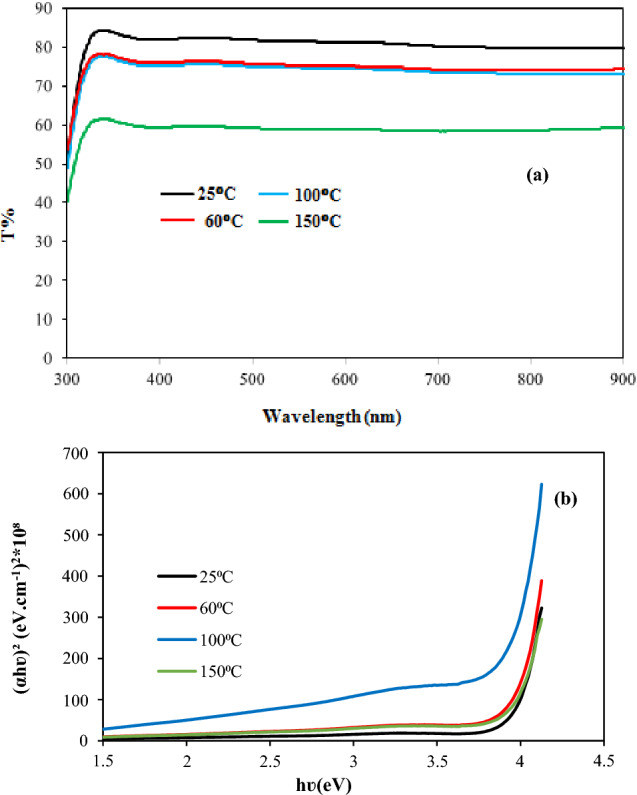
(**a**) Optical transmission plot and (**b**) (αhν)^2^ against photon energy of BaTiO_3_ films deposited at various substrate temperatures.

Figure 9a shows the effect of substrate temperature on the optical transmission of the BaTiO3 film. The optical transmittance of the film decreases as the substrate temperature increases due to the increased thickness and RMS surface roughness of the film with increasing substrate temperature. The average optical transmission of the films deposited at Ts  = 25, 60, 100, and 150 °C was 85%, 78%, 76%, and 59.5%, respectively. The transmission of the film deposited at 150 °C has decreased remarkably due to the increase in surface roughness and the formation of droplets and particulates^29,30^, which increases the scattering losses, and these agree well with AFM and SEM results.

The optical transmission of the films increases significantly after 352 nm and then tends to saturate, with a small blue shift detected for the sample deposited at Ts = 100 °C. The optical energy gap (E_g_) of the BaTiO_3_ film was calculated using the following Tauc's formula:

4 $$(\alpha h\nu {)}^{2}=A\left(h\nu -{E}_{g}\right)$$

where A is a constant depending on the material, αα is the absorption coefficient, hν is the photon energy. The value of Eg can be obtained by plotting (αhν)^2^ against hν and extrapolating the linear portion of the curve to the photon energy axis, as shown in Fig. 9b. The optical band gap of the BaTiO_3_ films deposited at 25, 60, 100, and 150 °C was 3.94 eV, 3.91 eV, 3.87 eV, and 3.84 eV, respectively.

**Figure 10:**
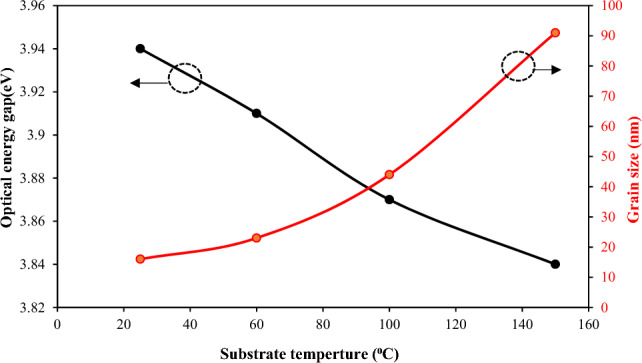
Dependence of optical energy gap of film on substrate temperature.

The obtained values of the energy gap are larger than that of bulk BaTiO_3_ (3.2 eV) due to the quantum size effect, and they are also in good agreement with reported data^28,30,31^. This means that the energy gap of the film decreases as the substrate temperature increases, as shown in Fig. 10. We can attribute the decrease in the optical energy gap with substrate temperature to an increase in the grain size of the film with increasing Ts, as previously shown in the SEM results.

**Figure 11:**
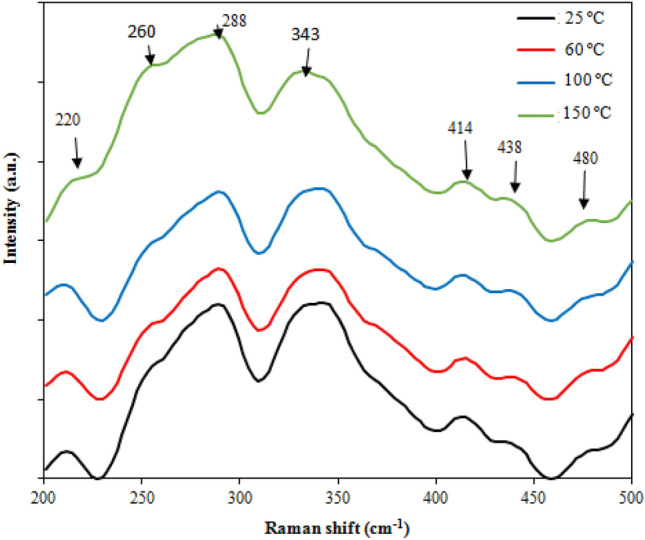
Raman spectra of BaTiO_3_ films prepared at different deposition temperatures.

Raman spectra of the BaTiO_3_ film deposited at different substrate temperatures are depicted in Fig. 11. As shown, the Raman band at 220 cm^−1^ is indeed the E (LO) (longitudinal optic) vibration mode. Raman peaks located at 260, 288, and 480 cm^−1^ are related to the A (TO) vibration mode, and the peaks observed at 414, 438 cm^−1^ belong to the E (TO)/E(OL) mode. The peak at 343 cm^−1^ is assigned to E (LO) + TO, B1) mode. The broad peaks give an indication of the polycrystalline nature of the deposited films and the degree of crystallinity. As reported, these obtained vibration modes arise from the displacement of Ti^4+^ ions brought on due to the effect of heating the substrate at high temperatures^32–35^. The full width at half maximum (FWHM) of the Raman bands was found to increase with increasing substrate temperature, and this could be correlated to the film's crystallinity quality.

**Figure 12:**
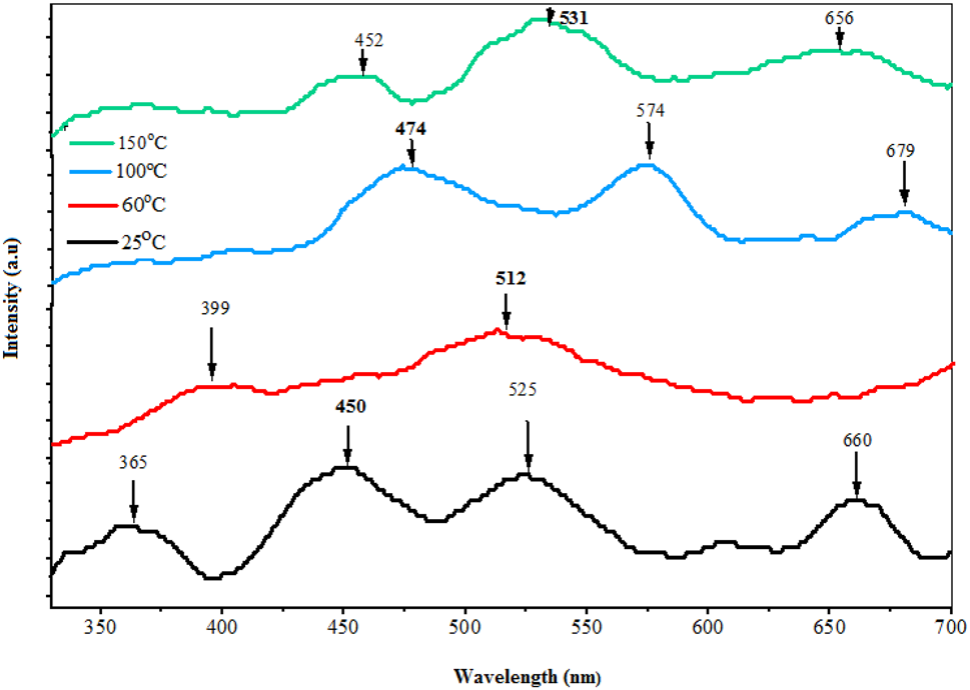
Room temperature PL spectra of BaTiO_3_ films deposited at different substrate temperatures.

Figure 12 illustrates the photoluminescence (PL) spectra of BaTiO_3_ films deposited on glass substrates at varying substrate temperatures, utilizing an excitation source with a 320 nm wavelength. At a deposition temperature of 25 °C, the film displays four broad emission peaks at 365 nm (3.39 eV), 450 nm (2.75 eV), 525 nm (2.36), and 660 nm (1.87 eV). The first emission peak is due to band-to-band transition and the third and fourth peaks are originated from magnetic-dipole transition^36^. Conversely, films deposited at substrate temperatures of 60 °C, 100 °C, and 150 °C exhibit prominent emission peaks at 512 nm (2.42 eV), 474 nm (2.61 eV), and 531 nm (2.33 eV), respectively. The other emission peaks can be attributed to the structural defects, deep level emission, and traps in the energy level system of the film as well as due to the small off-stoichiometry of the deposited films. The origin of blue emission is linked to delocalized electronic levels proximate to the valence and conduction bands, while the green emission results from the radiative recombination of an electron in the deep oxygen vacancy energy band with a hole in the valence band^37,38^. The intensity of PL emission peaks increases with substrate temperature due to the improvement in crystallinity of the films and a reduction in the optical band gap are responsible for the increase in visible emission intensity that is seen between 25 and 150 °C.

**Figure 13:**
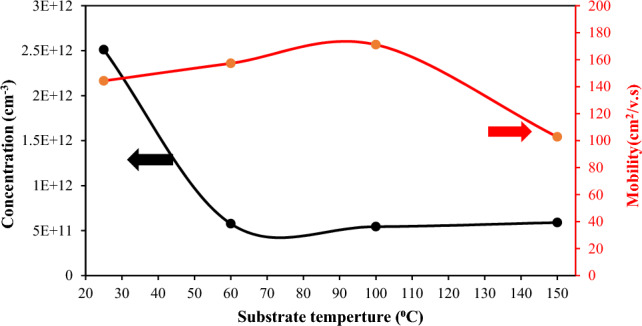
Carrier concentration and mobility of BaTiO_3_ film against substrate temperature.

**Figure 14:**
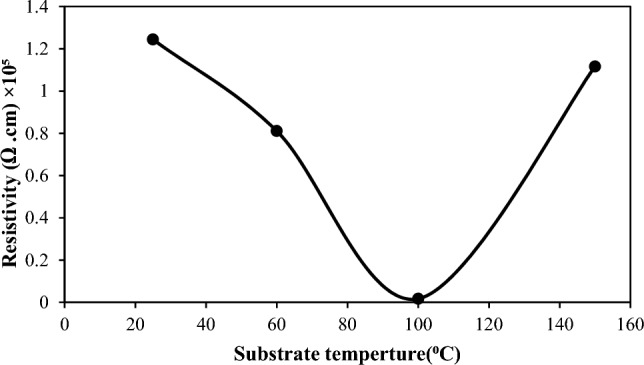
Electrical resistivity as a function of substrate temperature.

The electrical characteristics of the BaTiO_3_ films, including carrier concentration, mobility, electrical resistivity, and conduction type, were examined by Hall effect. The results reveal that the Hall coefficient of the films was negative, indicating that the films are n-type. The variation of the mobility and carrier concentration of the film with substrate temperature is shown in Fig. 13. The mobility of the film increased from 144 to 171 cm^2^/V·s as the substrate temperature rises from 25 to 100 °C as a result of increasing grain size and decreasing grain boundaries and decreased after this temperature due to the formation of droplets and particulates. The carrier concentration of the electrons decreases as the substrate temperature increases due to increasing mobility. Figure 14 illustrates that the electrical resistivity of the film decreases when the substrate temperature rises from 25 to 150 °C; it decreases from 1.24 × 10^5^ to 0.0169 × 10^5^ Ω cm due to the increasing grain size and electron mobility of the film^39–42^.

**Figure 15:**
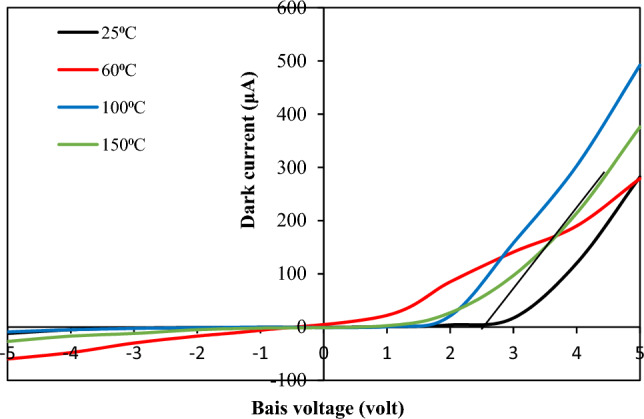
Dark I–V characteristics of BaTiO_3_/Si heterojunctions deposited at various substrate temperatures.

Figure 15 shows the dark I–V characteristics of the n-BaTiO_3_/p-Si heterojunction measured over the voltage range of − 5 to + 5 V. All fabricated heterojunctions demonstrate rectification properties, and the rectification factor was enhanced after increasing the substrate temperature to 100 °C due to the decreasing surface leakage current and increasing forward current as a result of reducing the electrical resistivity of the film. The forward current increases exponentially with bias voltage, and the substrate temperature affects the turn-on voltage (V_T_), ranging from 0.8 to 2.75 V depending on the electrical resistivity of the film. The forward currents of the heterojunctions deposited at 25, 100, and 150 °C show the domination of diffusion current, particularly at bias voltages larger than the turn-on voltage, while the heterojunction deposited at Ts  = 60 °C illustrates a forward current suffering from recombination processes due to structural defects and high series resistance.

The ideality factor (n) of the n-BaTiO_3_/p-Si heterojunction was calculated by using the following diode equation:

5 $$n=\frac{q}{{K}_{B}T}\frac{\Delta V}{ln\frac{\Delta I}{{I}_{s}}}$$

where q is the electron charge, k_B_ is the Boltzmann constant, and I_s_ is the saturation current of the heterojunction. The value of I_s_ was estimated by extrapolating the linear part of the semilogarithmic forward current–voltage characteristics to the current axis. The values of the ideality factor, turn-on voltage, and saturation current density as a function of substrate temperature are shown in Table 4. As shown in Table 4, the ideality factor is larger than unity, and the best value has been obtained for the heterojunction deposited at 100 °C, indicating that the BaTiO_3_-Si interface has a low density of surface states and traps^43–47^.

**Table 4:** Effect of substrate temperature on V_T_, Js, and n of the BaTiO_3_/Si heterojunction.

Ts (°C)	V_T_ (V)	Js (nA/cm^2^)	n
25	1.2	570	4.3
60	3	450	3.8
100	1.8	150	2.8
150	2.5	630	5

**Figure 16:**
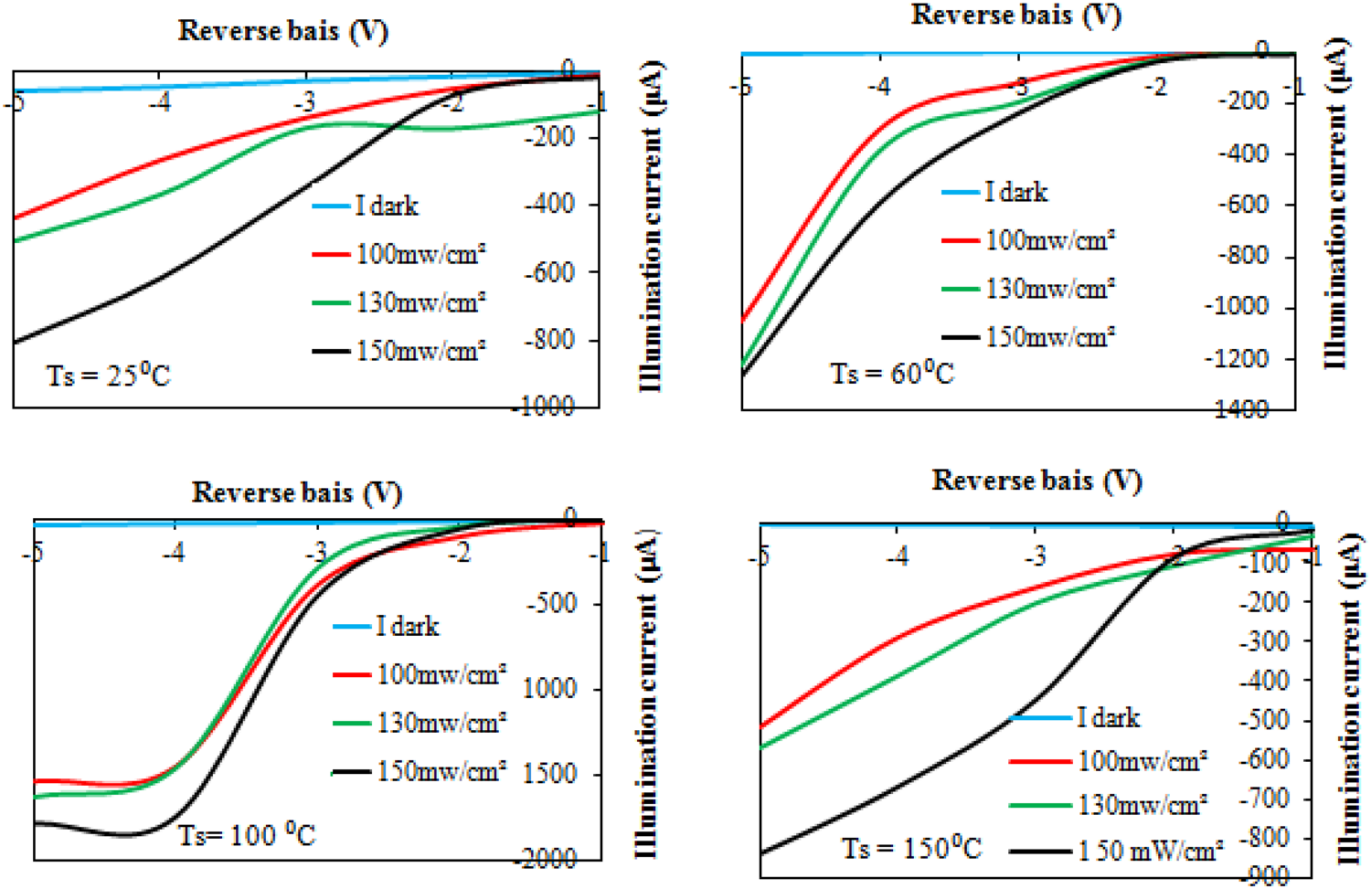
Illuminated I-V characteristics of n-BaTiO_3_/p-Si photodetectors.

**Figure 17:**
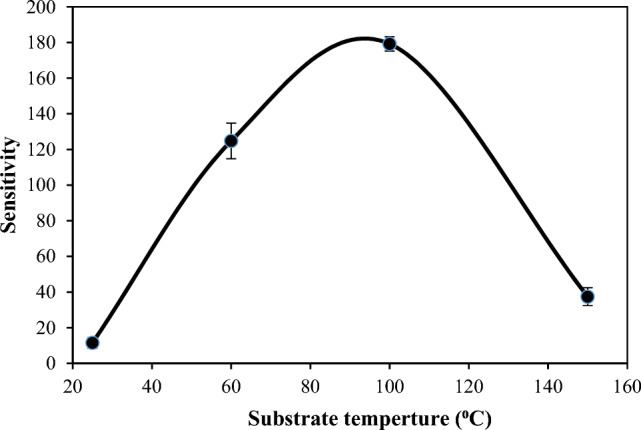
Impact of substrate temperature on sensitivity of BaTiO_3_/p-Si photodetector.

Figure 16 illustrates the I-V characteristics of the BaTiO_3_/Si heterojunction when the photodetector is illuminated with white light of varying intensities (100, 130, and 150 mW/cm^2^). The generation of electron–hole (e–h) pairs causes the photodetector’s current to increase when illuminated with light due to the absorption of light in the depletion region of the heterojunction. The increasing number of photogenerated e–h pairs as a result of increasing the light intensity leads to an increase in photocurrent as light intensity increases. No saturation in the photocurrent was detected after increasing the light intensity, indicating that the photodetectors have linearity properties. As depicted, raising the substrate temperature to 60 and 100 °C results in an increase in photocurrent due to enhanced film mobility and widening of the depletion region. However, an increase in substrate temperature to 150 °C leads to a decrease in photocurrent, attributed to nanoparticle agglomeration, the presence of droplets, and particulates. The sensitivity of the photodetectors as a function of substrate temperature is shown in Fig. 17, and the maximum sensitivity was around 180 for the photodetector deposited at Ts = 100 °C. We can attribute this result to the improvement of the junction characteristics with minimal structural and interface defects, as well as an increase in the diffusion length (L_D_​) of the minority carriers. The diffusion length increases as the mobility (μ) increases according to the following relationship:

6 $${L}_{D}=(\frac{KT}{q}\mu \tau {)}^{0.5}$$

where τ is the minority carrier lifetime of the photodetector and T is the operating temperature.

**Figure 18:**
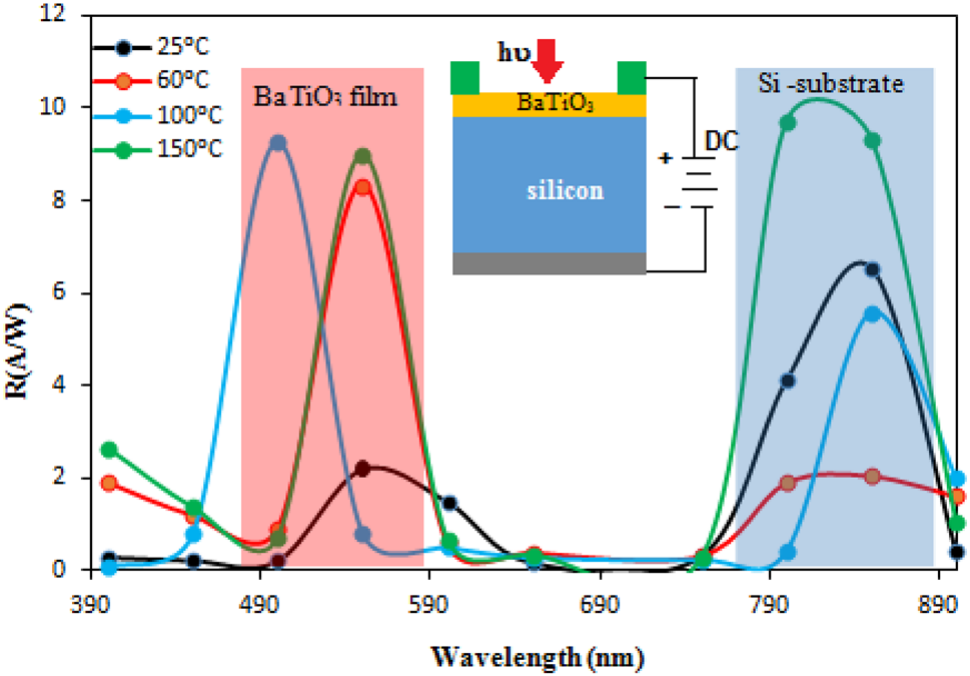
Spectral responsivity of BaTiO_3_ /p-Si heterojunction photodetectors prepared at various substrate temperatures at bias voltage of -5V. Inset is schematic diagram of the photodetector under reverse bias with electrical configuration.

The spectral responsivity of BaTiO_3_ /p-Si heterojunction photodetectors fabricated at different substrate temperatures is shown in Fig. 18. The photodetector's responsivity R_λ_ was calculated from the following equation:

7 $$\mathfrak{R}=\frac{{I}_{ph}}{P}$$

where P is the light power and I_ph_ is the photocurrent. We have observed two response peaks for all fabricated photodetectors. The photodetector deposited at room temperature exhibits two peaks located at 550 nm and 850 nm with responsivity values of 2.2 and 6.5 A/W, respectively. The first peak belongs to light absorbed in the depletion region located in the BaTiO_3_ (absorption edge of BaTiO_3_), and the origin of the second peak is the absorption edge of the silicon substrate^48–50^. Considering the absorption edge of nanostructured BaTiO_3_, the peak response should be around 350 nm rather than 550 nm. The absorption coefficient at 350 nm for the film is significantly higher compared to the wavelength of 550 nm, and the corresponding absorption depth is very small. This suggests that the 350 nm wavelength will be absorbed on the film surface, and the photogenerated carriers can rapidly recombine due to the presence of surface states and dangling bonds on the surface of the nanoparticles. Increasing the substrate temperature results in a significant enhancement of responsivity, accompanied by an observed shift in peak response. The responsivity of the photodetector increased from 2.2 A/W at 550 nm to 8.3, 9, and 9.2 A/W after increasing the substrate temperature to 60, 100, and 150 °C, respectively. This improvement in responsivity after increasing the substrate temperature is attributed to the enhancements in the structural, optical, and electrical properties of the film^51,52^.

The shift in peak of response after increasing the temperature to 100 °C can be attributed to the increase in the depletion layer thickness moving toward the BaTiO_3_ film and quantum size effect^53^, thereby enhancing the detection of short wavelengths. The presence of bias voltage plays a crucial role in increasing the responsivity by preventing e–h recombination and widening the depletion region width^54–56^.

**Figure 19:**
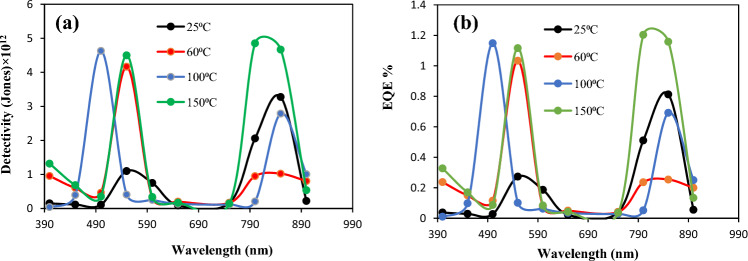
(**a**) D*and (**b**) EQE of n-BaTiO_3_/p-Si heterojunction photodetectors prepared at various substrate temperatures.

The effect of the substrate temperature on the specific detectivity of the photodetector is demonstrated in Fig. 19a. The specific detectivity (D*) of the photodetector is defined as the minimum detectable power and it can be estimated by the following formula:

8 $$\mathrm{D }* ={R}_{\uplambda }{(A/2q{I}_{d})}^\frac{1}{2}$$

here, A is the sensitive area of the photodetector, and I_d_ is the dark current. The highest value of D^∗^ was 4.62 × 10^12^ Jones at 500 nm for the photodetector prepared at Ts  = 100 °C and corresponded to a noise equivalent power (NEP) of 0.21 PW. Figure 19b illustrates the external quantum efficiency (EQE) of the photodetector deposited at different substrate temperatures. As shown in Fig. 19b, the photodetector fabricated at 100°C has EQE exceeding unity, and this result is due to light trapping, minimal structural defects, minimal grain boundaries, widening the depletion layer thickness, low density of surface states, and recombination^57–59^. Table 5 provides a comparison of the figures of merit for the BaTiO_3_/Si photodetector deposited at 100 °C with those of heterojunctions based on silicon photodetectors reported in the literature.

**Table 5:** Figures of merit of some heterojunction-based silicon photodetectors compared with fabricated BaTiO_3_/Si at 100 °C (best photodetector).

Photodector type	Preparation method	Peak wavelength (nm)	Responsivity R_λ_ (A/W)	External quantum efficiency EQE %	Detectivity D* (Jones)	References
Bi_2_O_3_/Si	Laser ablation in liquid	610	0.23	0.5 × 10^2^	1.5 × 10^11^	^50^
PbI_2_/Si	pulsed laser deposition	610	0.4	0.5 × 10^2^	20.4 × 10^12^	^51^
ZnO/Si	Laser ablation	450	9.9	2.951 × 10^2^	4.3 × 10^12^	^60^
HgI_2_/Si	pulsed laser deposition	400	1.09	3 × 10^2^	3.6 × 10^12^	^61^
This work	pulsed laser deposition	500	9.2	1.14 × 10^2^	4.62 × 10^12^	

**Figure 20:**
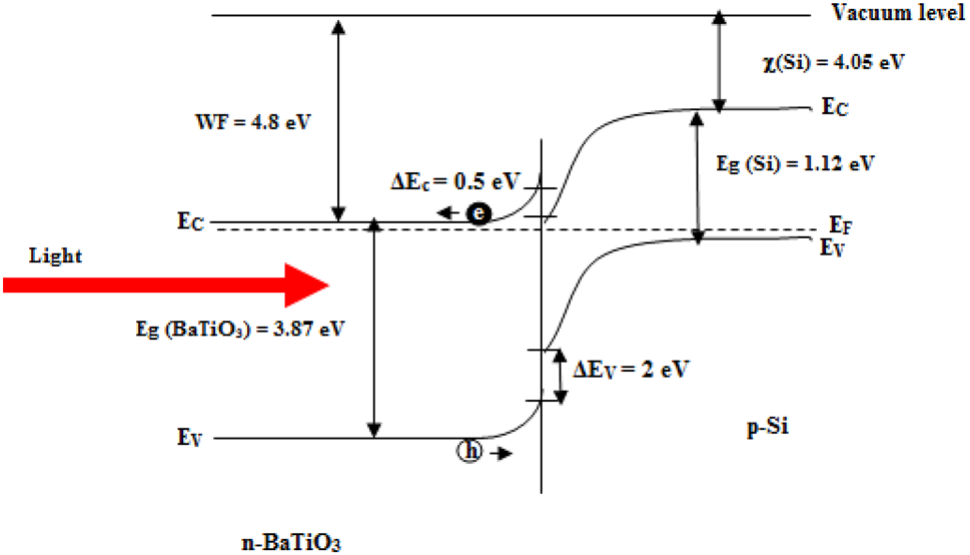
Illuminated energy band diagram of BaTiO_3_/Si heterojunction photodetector prepared at 100 °C.

To understand the mechanism of light detection in BaTiO_3_/Si photodetector, the energy band diagram was constructed, as shown in Fig. 20. The conduction band offset between the BaTiO_3_ nanostructure and silicon substrate was estimated as ΔE_C_ = χ_BT_ − χ_Si_ (4.8–4.05) = 0.75 eV, and the valence band offset as ΔE_V_ = (Eg_BT_ − Eg_Si_ ) – (χ_BT_ – χ_CdS_) = (3.87 – 1.12) – 0.75 = 2 eV. Upon the light incident on the BaTiO_3_ film, it will be absorbed in the depletion region of the n-BaTiO_3_, which leads to the generation of an e–h pair and an consequently increased the photocurrent of the photodetector. The presence of the internal electric filed as a result of intimate contact between BaTiO_3_ film and silicon substrate prevents e–h pair recombination^62,63^ The light exciting the electron from valence band to conduction band produces a free electron in conduction and a free hole in valence band, and these carriers conduct current in photodetector.

## Conclusions

In this study, high-performance n-BaTiO_3_/p-Si heterojunction photodetector using the laser deposition route was successfully fabricated without using buffer layer. The structural, optical, and electrical properties of BaTiO_3_ was studied as a function of substrate temperature. X-ray diffraction studies confirmed that nanostructured BaTiO_3_ was polycrystalline with a mixture of tetragonal and hexagonal phases. The particle size increases with an increase in the substrate temperature. The optical energy gap of the film decreased from 3.94 to 3.84 eV as Ts increases from 25 to 100 °C due to an increase in the grain size. AFM investigation confirms that the surface roughness of the film increases as the substrate temperature increases, enhancing the light trapping of the film. Based on the obtained results, the optimal substrate temperature was found to be 100 °C. Examining the impact of substrate temperature on the BaTiO_3_/Si photodetector's figures of merit, we observed a superior device at 100 °C with a responsivity of 9.2 A/W, a specific detectivity of 4.62 × 10^12^ Jones, and an external quantum efficiency of 114%. Furthermore, we constructed the illuminated energy band diagram for the n-BaTiO_3_/p-Si heterojunction photodetector to understand the photodetection mechanism.

Collectively, our results emphasize the potential of these photodetector for applications requiring the detection of very weak light signals, affirming its effectiveness in specialized and demanding environments.

## Data Availability

The datasets generated during and/or analyzed during the current study are available from the corresponding author (R.A. Ismail)) on reasonable request.
